# Supplementary material for: Sexual Dimorphism in Metabolic Responses to Western Diet in Drosophila melanogaster
Source: Biomolecules. 2021 Dec 27;12(1):33. doi: 10.3390/biom12010033 (PMC8774106; doi:10.3390/biom12010033)
Supplement: Supplementary file 1 [file biomolecules-12-00033-s001.zip › biomolecules-1498228-supplementary.pdf]

biomolecules

MDPI

Article

Sexual Dimorphism in Metabolic Responses to Western Diet in *Drosophila melanogaster*

Sofie De Groef [1,*], Tom Wilms [1], Séverine Balmand [2], Federica Calevro [2] and Patrick Callaerts [1,*]

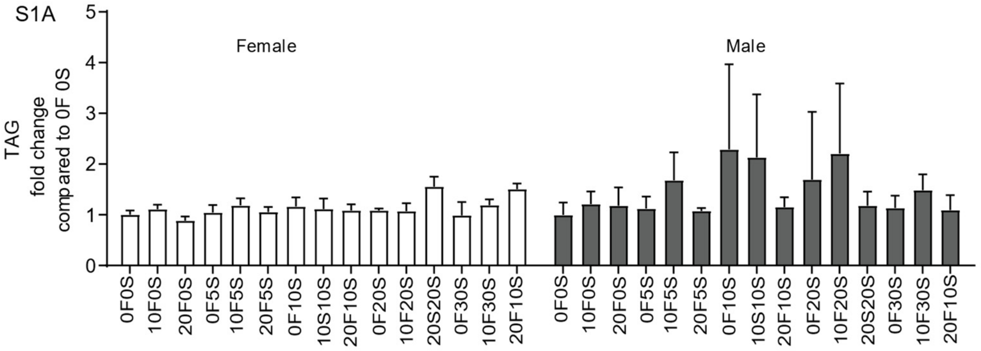

Source of Variation	% of total variation	P value	P value summary	Significant?	
I nteraction	14.05	0.0529	ns	No	
diet	13.81	0.0584	ns	No	
sex	5.933	0.0016	**	Yes	
ANOVA table	SS (Type Ⅲ)	DF	MS	F (DFn, DFd)	P value
I nteraction	7.995	14	0.5711	F (14, 115) = 1.762	P=0.0529
diet	7.859	14	0.5614	F (14, 115) = 1.732	P=0.0584
sex	3.376	1	3.376	F (1, 115) = 10.41	P=0.0016
Residual	37.28	115	0.3241		

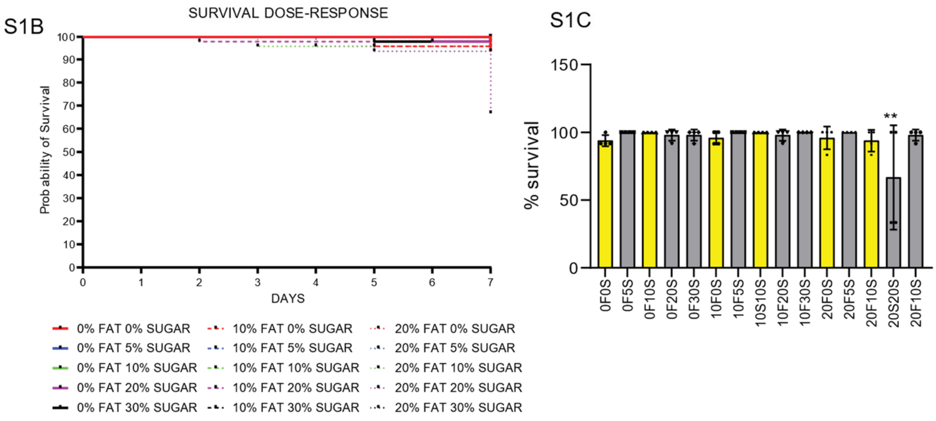

Supplementary Figure S1. Triglycerides (TAG) and survival of flies on diets supplemented with the increasing concentration of sugar, fat, or a combination of both. **(A)** Fold changes of the TAG level in adult male and female *Dahomey* flies exposed to the normal diet (control diet (CD)), fat (coconut oil)-supplemented diet (FD1 and FD2), (sucrose) sugar-supplemented diet (SD), and western diets (WD1 and WD2). The table depicts the two-way ANOVA analysis of the data in the table. **(B)** Kaplan–Meier survival curves for 12 male and 12 female flies transferred to two vials each of the 15 dietary conditions. The number of flies of each sex that died was determined every day for 7 days. At the end of the 7-day period, the deaths of flies were the result of sticking into the food containing high amounts of fat. **(C)** Bargraph displays the data from Kaplan Meier survival curves as percentage of survival for each of the 24 flies (12 males, 12 females) on the 15 dietary conditions. Yellow bars represent the dietary conditions that were used as selected dietary conditions. Condition

20F20S displays a significant decrease in survival. Since this is an artefact of flies trapped in the food, these data were excluded from the One-Way ANOVA, F = 1.230, p = 0.293).

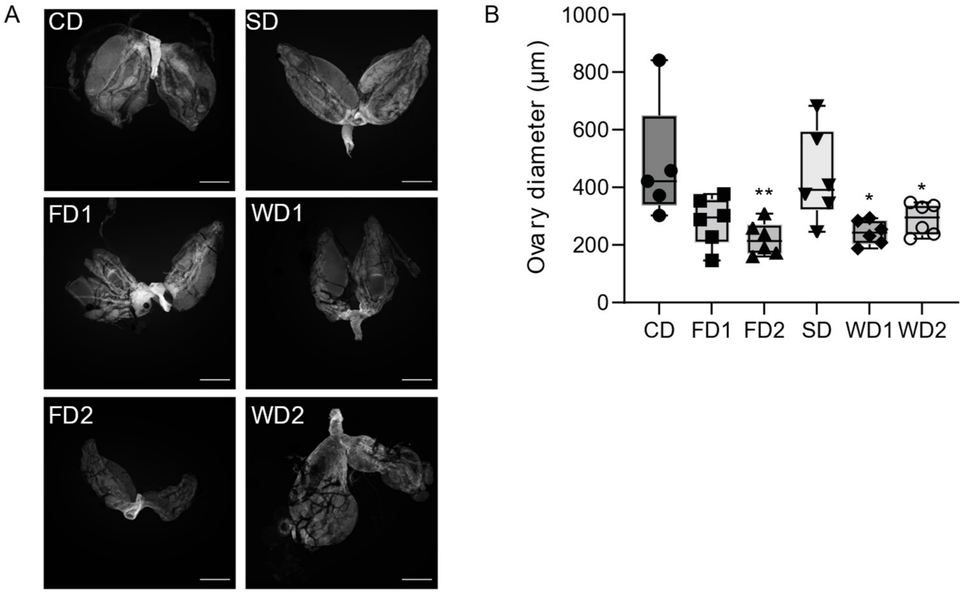

Supplementary Figure S2. **(A)** Rhodamin phalloidin staining of dissected ovaries from female *Dahomey* flies exposed to CD, fat (coconut oil)-supplemented diet (FD1 and FD2), (sucrose) sugar-supplemented diet (SD), and western diets (WD1 and WD2). Scale bars correspond to 200 μm **(B).** Graph displaying the average ovary diameter of ovaries from the flies with the diets. The data are expressed as an average diameter of ovaries. (one-way ANOVA, F = 4.815, $p = 0.0025$) with a Dunnett's multiple comparisons test comparing the data with the diets to those with the CD per sex (* $p < 0.05$, ** $p < 0.005$).

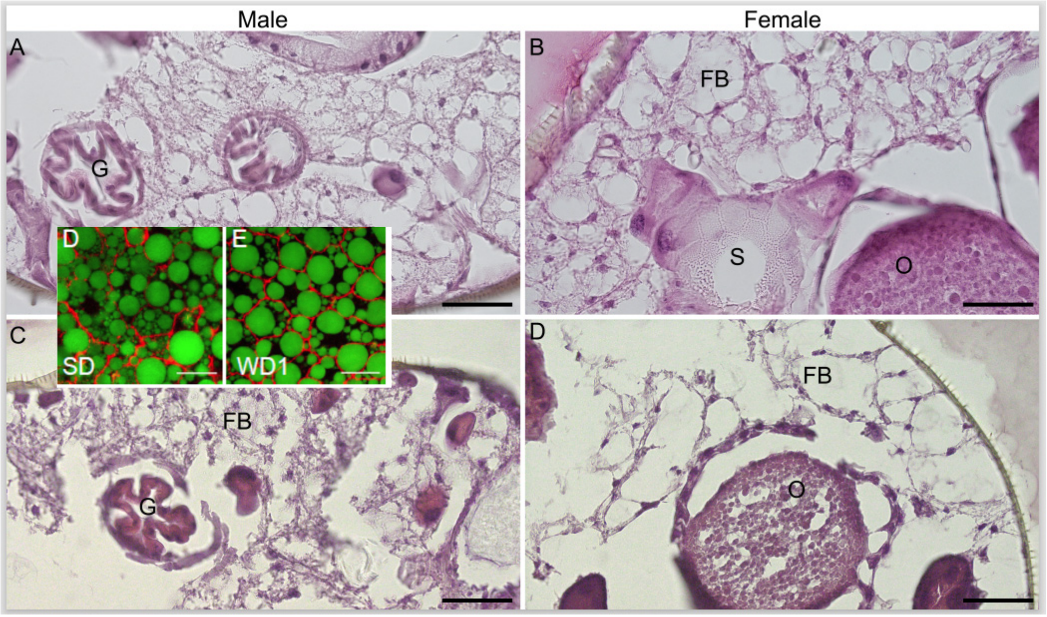

Supplementary Figure S3. Histological analysis of female or male flies exposed to western diets. The cross-sections of the abdomens of the control flies showed a classic organization of the fat body in both males (**A**) and females (**B**). This organization was altered upon the exposure of flies to the western diet (WD1). Males (**C**) were less affected than females (**D**). Abbreviations: FB, fat body; G, Gut; S, stomach; O, oocyte. Scale bars represent 20 μm.
